# Prevalence and distribution of musculoskeletal disorders causing unfitness for military service among young adult men: An epidemiologic study

**DOI:** 10.12669/pjms.311.5674

**Published:** 2015

**Authors:** Şafak Ekinci, Necmettin Kocak, Ibrahim Aydin, Kenan Koca, Ramazan Akyildiz, Omer Ersen, Selim Kilic

**Affiliations:** 1 Şafak Ekinci, Agri Military Hospital, Agrı, Turkey.; 2 Necmettin Kocak, Turkish Coast Guard Command, Ankara, Turkey.; 3 Ibrahim Aydin, Sarikamis Military Hospital, Kars, Turkey.; 4 Kenan Koca, Gulhane Military Medical Academy, Department of Orthopedic Clinic, Ankara, Turkey.; 5 Ramazan Akyildiz, Ministry of Defence, Ankara, Turkey.; 6 Omer Ersen, Erzurum Military Hospital, Erzurum, Turkey.; 7 Selim Kilic, Gulhane Military Medical Academy, Department of Public Health, Ankara, Turkey.

**Keywords:** Unfitness to military service, Musculoskeletal disorders, Fracture sequel

## Abstract

**Objectives:**

The aim of this cross-sectional epidemiologic study was to investigate the prevalence and distribution of musculoskeletal disorders causing unfitness to Turkish Military Service.

**Methods:**

This study has been carried out by examining the medical reports of 1.777.500 people who applied to the Turkish Armed Forces for military service between 2009-2011. Age and geographic region of individuals were compiled and organized in groups. Musculoskeletal disorders were classified mainly as fracture sequel, spine disorders, absence of phalanges, extremity amputation, aggressive or multiple benign tumors of bones and pes planus.

**Results:**

Unfitness to military service caused by musculoskeletal disorders was found to be 6.53‰ in 2009, 7.10‰ in 2010 and 7.28‰ in 2011. The prevalence of musculoskeletal diseases has increased by years. The prevalence of fracture squeal by years was found to be 2.83‰ in 2009, 3.10‰ in 2010 and 3.03‰ in 2011. In this study, the most common musculoskeletal disorders were: limitation of joint mobility (0.89‰), degeneration of joint surface (0.69‰), lower and upper limb discrepancies (0.60‰), posterior fusion surgery (0.59‰) and the absence of the phalanges in hand (0.51‰). We found an increase in both the prevalence of posterior fusion surgery and the absence of the phalanges in study group.

**Conclusion:**

These results has given information about severe musculoskeletal disorders among young adult male in Turkey. New studies including young adult female will add important information to our knowledge about musculuskelatal problems in our community.

## INTRODUCTION

The occurrence of musculoskeletal disorders in the community may be congenital or due to acquired etiologies. Musculoskeletal disorders are the main cause of disability in adults and are one of the most significant parameters to understand the level of public health.^1^

In Turkey, military service is compulsory for men.^2^ Every man has to undergo a medical examination as specified in Health Ability Regulation (HAR) of Turkish Armed Forces (TAF) before starting his military service. Military service has not to be done by those with health problems diagnosed in their medical examinations that may cause be inconvenient for military service. As a result, these data gives detailed information about the major health problems among young adult males in Turkey.

This study was conducted to investigate the prevalence and distribution of the cases to be determined as unfitness for military service due to musculoskeletal disorders among young adults who underwent medical examinations for compulsory military service between the years 2009-2011 in Turkey.

## METHODS

***Study Group: *** A cross-sectional prevalence study was carried out by examining the medical reports of 1.777.500 people who applied to TAF for military examination between 2009 and 2011. The numbers of young adult males used in prevalence calculation were 579.503 in 2009, 583.299 in 2010 and 614.698 in 2011. 

***Administrative and Ethical Permissions: ***Data for this study have been obtained after obtaining the necessary administrative permits from the Surgeon General Office of Ministry of National Defense. Required ethical permissions of the study have been obtained from Ethics Committee of Gulhane Military Medical Academy (GMMA).

***Descriptions in the Survey: ***Military service is a statutory obligation in for each male person who is twenty years old. These persons are enlisted to TAF if they have no reason for postponing it, such as higher education or a medical condition. However, individuals having serious medical problems do not register to military service. According to the HAR of TAF, these unfit persons are described by a code from the following classification:

*Code A:* those fit for military service, 

*Code B:* those unfit for peacetime but that can be drafted in wartime, 

*Code C:* those in treatment and in recovery 

*Code D:* those unfit for peacetime and wartime. 

For the study, only the diseases that constitute complete unfitness for the military service (B and D) were taken into account in calculating the prevalence, whereas those fit for military service and those in treatment and in recovery (A and C) were excluded from the study.

Candidates for military service were examined by two medical doctors in military councils at recruiting stations, which are placed in provincial or subprovincial centers. Diagnoses of certain disorders such as phalanx absence in hand and amputation can be done during these examinations. If further medical investigation and radiological tests were needed, the candidate was referred to a military hospital to evaluate whether he was unfit for military service. Detailed physical examination and plain radiography were frequently used to evaluate candidates. Big Goniometer was used to measure the range of joint motion. In addition, computerized tomography (CT) and magnetic resonance imaging (MRI) were used when necessary.

***Evaluating the data and statistical analysis: ***In order to examine the geographic distribution, the provinces where the recruiting offices of the cases were grouped as West, South, Central, Northern and Eastern zoning system prepared by Turkish Statistical Institute.^3 ^Since medical examinations before military service began at the ages of 19-20, the ages of the cases were grouped as “19, 20, 21-24, and 25 years and above” when examining the demographic characteristics.

Musculoskeletal disorders were classified as fracture sequel, spine disorders, absence of phalanges and amputation, and other disorders (Table-II). Limitation of range of motion, degeneration of joint surface, lower and upper limb discrepancies, deformities of long bones, varus-valgus-recurvate deformities of joint and limitation of inversion-eversion range of subtalus joint caused by fracture were included in the fracture sequel group. Posterior fusion surgery of vertebrates, kyphoscolliosis, limitation of spine motion and lumber disc surgeries were included in the spine disorders group. Pes planus, malign and multiple benign bone tumors were included in the others group.

Data was collected from health reports belonging to the candidate conscripts who are liable for the compulsory military service but unfit for it which were kept in Health Directorate of the Ministry of National Defense. Descriptive statistics for discrete data, namely frequency, percentage, mean ± standard deviation for continuous variables were used using the SPSS 15.0 program.

## RESULTS

Health data of 1.777.500 military service obligors whose health examinations have been carried out between the years 2009-2011 has been reviewed in our study and it was found that 12,368 cases have musculoskeletal diseases. The mean age was 22.8±6.3 years in our study group. The prevalence of musculoskeletal by years was found to be 6.53‰ for 2009, 7.10‰ for 2010 and 7.28‰ for 2011. Thus, the prevalence of musculoskeletal diseases has increased.

In our study, it was found that the most populous age group was 19 (35.3%) and the most crowded region in terms of the musculoskeletal disorders is the Eastern Anatolia Region (35.9%) (Table-I).

**Fig.1:**
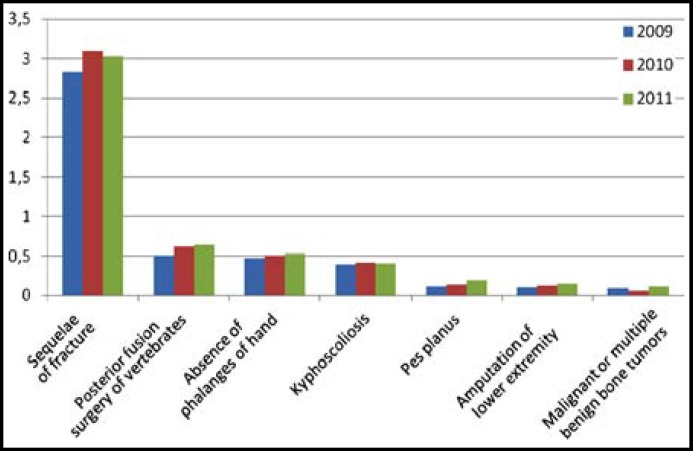
The change in prevalence of the most likely diagnoses between 2009-2011 (per thousand).

In our study, the decision for unfit for military service has been taken for 39.3% of the cases treated for musculoskeletal system as a result of fracture sequelae. The prevalence of fracture sequelae by years was found to be 2.83‰ in 2009, 3.10‰ in 2010 and 3.03‰ in 2011. In this study, the most common five musculoskeletal disorders were: limitation of joint mobility (0.89‰), degeneration of joint surface (0.69‰), lower and upper limb discrepancies (0.60‰), posterior fusion surgery (0.59‰) and the absence of the phalanges in hand (0.51‰) (Table-II). The changes in the prevalence of diseases by years are shown in Fig.1. An increase in both the prevalence of posterior fusion surgery and the absence of the phalanges in hand was noticed.

There was no statistical comparisons. So no p value was given.

## DISCUSSION

Musculoskeletal diseases are one of the major causes of disability around the world.^4^ In this study, the prevalence of musculoskeletal disorders by years has been found to be 6.53‰ in 2009, 7.10‰ in 2010 and 7.28‰ in 2011. Kilic found a prevalence of musculoskeletal disorders of 6.94 per every thousand people in a similar study that he had carried out on young adult males in Turkey in 1999.^5^ A prevalence of musculoskeletal disorders of 41.1 per thousand was found in a study that Taanila conducted on the people on the period of recruitment.^6^ In the study that Lawrence et al have carried out in the United States of America (USA), it was found that the musculoskeletal diseases affected approximately 15 % of the society (40 million) in 1995 and it was predicted that 18.2% of the society (59.4 million) will be affected by 2020.^7^ Salaffi et al. reported that the overall prevalence of musculoskeletal conditions in the general adult population was 26.7% and being significantly higher among women than men in Italy.^8^ They also found that disease prevalence increased significantly with age. Reynolds et al. expressed that the estimated prevalence of disability in adults due to musculoskeletal diseases has been 50.1 per thousand in Canada. It is observed that the prevalence is 6.2 per thousand in the people between the ages of 15-24, and that in individuals over 85 years of age it rises to 264.7 per thousand.^9^ In this study, it is observed that the prevalence of musculoskeletal diseases has been lower than in the studies carried out in the USA and Italy and similar to the prevalence value in young adult males in the study carried out in Canada and Turkey, and that the prevalence has increased to 2009 to 2011. The most likely explanation for the low-prevalence here described is that young males were studied. The increase in musculoskeletal disorders from 2009 to 2011 suggests that occupational accidents, traffic accidents and sports injuries may have been increasing in the society in the recent years.

Limited range of joint motion, degeneration of joint surface, deformities of long bones and extremity discrepancies and majority of the varus - valgus - recurvatum deformities of joints constitute secondary complications to the fracture sequel. These fractures occur as a result of traffic accidents, sports injuries, gunshot wounds, and high falls. Some of the limitations of joint motion may develop secondary to rheumatic diseases (RA, JRA). Degeneration of joint surface may also occur as a result of perthes disease, avascular necrosis of the femoral head, necrosis of the lunate (*Kienböck's* disease) and JRA. In addition, depending on the extension of cartilage lesions, disturbance of joint surface may result from sports injuries. Atrophy in thigh may develop due to instability of knee and ankle joints. Circumference difference of extremities may result from congenital or acquired diseases causing hemihypertrophy, phlebitis, or major venous insufficiency. The prevalence of occurring diseases depending on the fracture sequel is shown in Table-II. Sequels of fracture prevalence among young adult males between 2009 and 2011 have been found to be 3.00‰. 

Spinal disorders that interfere with military service may be scoliosis, kyphosis or kyphoscoliosis. It is stated that the prevalence of vertebral anomalies in newborns worldwide is 0.5-1.0 per thousand.^10^ In Kilic’s study, the prevalence of vertebral anomalies is stated as 0.54 per thousand.^5^ Kyphoscoliosis is one of the most common abnormalities of the spine, with an estimated prevalence for mild deformity of 1 in 1000 people and for severe deformity of 1 in 10,000 people in the United States.^11^ In this study, the prevalence of kyphoscoliosis over the years has been found to be 0.39‰ for 2009, 0.42‰ for 2010, and 0.41‰for 2011. Posterior fusion surgeries have been frequently applied in the treatment of scoliosis and also to treat unstable vertebral fractures. In this study, the prevalence of vertebral posterior fusion surgeries by years has been found to be 0.51‰ for 2009, 0.62‰ for 2010, and 0.64‰ for 2011. It is found that the prevalence of posterior fusion surgery has increased in years. When the posterior fusion surgery and kyphoscoliosis diagnoses were evaluated together, it was observed that the prevalence was similar to the prevalence of these abnormalities in the world.

Majority of the phalanx absence in hand depends on traumatic amputations that occur as a result of occupational accidents. A very small subset of them are in the form of congenital absence or deficiency. In this study, the prevalence of the phalanx absence in hand by years is identified as; 0.47‰ for 2009, 0.51‰ for 2010, 0.53‰ for 2011. In Kilic’s study, the prevalence of the phalanx absence in hand has been stated as 0.22 per thousand.^5^ It is determined that the prevalence has approximately increased 2-2.5 times within years. This increase suggests that occupational accidents have increased in the society.

Pes planus is a common medical condition defined as flattening in the medial longitudinal arch of the foot during bearing weight and orthopedic practice.^12^ According to the HAR of TAF, mild flexible pes planus have code A and they enlist to military service. However, rigid pes planus and severe flexible pes planus have code B and they do not register to military service. For diagnosis of severe pes planus, we used calcaneal high angle and broken of first metatars-talus line in lateral foot radiography, talo-calcaneal separation in AP foot radiograph and tarsal degeneration in both radiographs. In our study, pes planus constitutes 2.1% of the cases examined in orthopedic clinic. The prevalence of pes planus by years has been found to be 0.11‰ in 2009, 0.13‰ in 2010 and 0.19‰ in 2011. The prevalence of pes planus has been found as 0.93‰ in Kilic’s study.^5^ Yucesan et al. reported that they have found a prevalence of pes planus of 2.28 per thousand people.^13^ In a study carried out on adolescents in Germany, the prevalence of foot deformity has been found as 13.7%. The prevalence of flexible pes planus has been found to be 6.2% and the prevalence of rigid pes planus has been identified as 0.5%.^14^ In our study, the prevalence of pes planus has been found to be lower than other studies. This lower prevalence results from the inclusion of rijid and severe flexigle pes planus as criteria of unfitness for military service.

The reasons of lower extremity amputations in our patients group may be either congenital anomalies or traumatic amputations depending on traffic accidents. The Global Lower Extremity Amputation Study Group has investigated amputation in Europe, North America and East Asia; the highest prevalence of amputation is found in the Navajo Nation in the United States (0.44 persons per thousand every year), and the lowest prevalence is found in Madrid (0.03 persons per thousand every year). It was determined that the amputation prevalence has increased with increasing age and is higher in males than in females.^15^ In this study, the prevalence of lower extremity amputation over the years has been found to be 0.10‰ in 2009, 0.12‰ in 2010 and 0.15‰ in 2011. Thus, the prevalence has increased over the years, is lower than the prevalence in the studies conducted in Turkey (0.29 per thousand) and Navajo (0.44 per thousand) and is similar to the values found in the studies conducted in Japan (0.12 per thousand) and Taiwan (0.09 per thousand).^5^^,^^15^^-^^17^

Most bone tumors develop in the first several decades of life, and often tend to develop in long bones.^18^ Bone cancer is a malignant tumor of the bone that destroys normal bone tissue. Not all bone tumors are malignant. In fact, benign (noncancerous) bone tumors are more common than malignant ones. Based on the most recent data, approximately 0.1 percent of men and women will be diagnosed with bone and joint cancer at some point during their lifetime.^19^ In this study, the prevalence of malignant, benign aggressive and multiple benign bone tumors by years has been found as 0.09‰ for 2009, 0.06‰ for 2010 and 0.11‰ for 2011. As to the Kilic’s study, the prevalence has been found to be 0.07‰.^5^ Prevalence is considered to be parallel to each other.

**Table-I:** Age and region characteristics of the patients with musculoskeletal disorders

		**2009**		**2010**		**2011**		**Total**	
		**n**	**%**	**n**	**%**	**n**	**%**	**n**	**%**
Age groups	19	1462	38.6	1407	34,2	1495	33,4	4364	35.3
	20	643	17.0	841	20.5	927	20.7	2411	19.5
	21-24	856	22.6	916	22,4	1123	25.1	2895	23.4
	25 and above	820	21,8	946	22,9	932	20.8	2698	21.8
Region	West	606	16,0	665	16,2	654	14,6	1925	15,6
	South	379	10,0	393	9,6	497	11,1	1269	10,3
	Central	1008	26,7	1048	25,5	1181	26,4	3237	26,2
	North	436	11,5	515	12,5	544	12,2	1495	12,1
	East	1352	35,8	1489	36,2	1601	35,8	4442	35,9
Total		3781	100,0	4110	100,0	4477	100,0	12368	100.0

**Table-II:** Prevalence of the diagnoses

Diagnoses	2009			2010			2011			Total		
	n	%	%_0_*	n	%	%_0_*	n	%	%_0_*	n	%	%_0_*
a. Sequelae of fracture	1642	41.7	2.83	1811	44.0	3.10	1862	41.6	3.03	5315	43.1	3.00
Limitation of range of motion of joint	476	12.6	0.82	552	13.4	0.95	553	12.4	0.90	1581	12.8	0.89
Degeneration of the joint surface	394	8.6	0.68	423	10.2	0.73	416	9.3	0.68	1233	10.0	0.69
Lower and upper limb discrepancies	330	8.8	0.57	379	9.3	0.65	350	7.7	0.57	1059	8.6	0.60
Deformities of long bones after fracture	220	5.8	0.38	208	5.1	0.36	271	6.1	0.44	699	5.7	0.39
Varus-valgus-recurvate deformities of the joints	94	2.5	0.16	79	1.9	0.14	98	2.2	0.16	271	2.2	0.15
Limitation of inversion-eversion range of subtalus joint	128	3.4	0.22	170	4.1	0.29	174	3.9	0.28	472	3.8	0.27
b. Spine disorders												
Posterior fusion surgery of vertebrates	295	7.8	0.51	362	8.8	0.62	392	8.8	0.64	1049	8.5	0.59
Degeneration of the joint surface Lower and upper limb discrepancies	225	6.0	0.39	243	5.9	0.42	251	5.6	0.41	719	5.8	0.41
Limitation of motion in the spine	66	1.7	0.11	87	2.1	0.15	88	2.0	0.14	241	1.9	0.14
Lomber disc surgeries	62	1.6	0.11	77	1.9	0.13	76	1.7	0.12	215	1.7	0.12
c. Absence of phalanges in hand and foot and amputations												
Absence of phalanges in hand	274	7.2	0.47	298	7.3	0.51	329	7.3	0.53	901	7.3	0.51
Absence of phalanges in foot	101	2.7	0.17	107	2.6	0.18	121	2.7	0.20	329	2.7	0.19
Amputation of lower extremity	58	1.5	0.10	72	1.8	0.12	90	2.0	0.15	220	1.8	0.12
Amputation of upper extremity	46	1.2	0.08	53	1.3	0.09	40	0.9	0.07	139	1.1	0.08
d. Other disorders												
Pes planus	66	1.7	0.11	75	1.8	0.13	116	2.6	0.19	257	2.1	0.15
Malignant or multiple benign bone tumors	51	1.3	0.09	36	0.9	0.06	65	1.5	0.11	152	1.2	0.09

* prevalence.

Limitations of our study were, although our sample size was very large and the medical reports were comprising for three years period, the study was conducted among young adult male and because of the aim of study we determined only severe musculoskleteal disorders.

## CONCLUSION

Musculoskeletal disorders commonly seen in young adult males who are unfit for military service in Turkey have been examined in this study. It is found that the most common musculoskeletal disorders causing unfitness for military service in young adult males were limitation of joint mobility, degeneration of joint surface, lower and upper limb discrepancies caused by fracture sequel. Because of the very large sample size and three years period time frame this study provide valuable addition to the medical literature about musculoskeletal disorders.
